# Purification, Identification, and Properties of a Novel Carotenoid Produced by *Arthrobacter* sp. QL17 Isolated from Mount Qomolangma

**DOI:** 10.3390/antiox11081493

**Published:** 2022-07-29

**Authors:** Xue Yu, Kan Jiang, Wei Zhang, Shuqing Dong, Yujie Wu, Gaosen Zhang, Shiyu Wu, Tuo Chen, Guangxiu Liu

**Affiliations:** 1 Key Laboratory of Desert and Desertification, Northwest Institute of Eco-Environment and Resources, Chinese Academy of Sciences, Lanzhou 730030, China; yuxue@lzb.ac.cn; 2 Key Laboratory of Extreme Environmental Microbial Resources and Engineering, Lanzhou 730030, China; wuyujie18@mails.ucas.ac.cn (Y.W.); zhanggaosen@nieer.ac.cn (G.Z.); 15619010897@163.com (S.W.); chentuo@lzb.ac.cn (T.C.); 3 University of Chinese Academy of Sciences, Beijing 100049, China; 4 Agronomy College, Gansu Agricultural University, Lanzhou 730070, China; jiangk19@126.com; 5 CAS Key Laboratory of Chemistry of Northwestern Plant Resources and Key Laboratory for Natural Medicine of Gansu Province, Lanzhou Institute of Chemical Physics, Chinese Academy of Sciences, Lanzhou 730030, China; sqdong@licp.cas.cn; 6 State Key Laboratory of Cryospheric Science, Northwest Institute of Eco-Environment and Resources, Chinese Academy of Sciences, Lanzhou 730030, China

**Keywords:** *Arthrobacter*, carotenoid, antioxidant, anticancer

## Abstract

The genus *Arthrobacter* is a source of many natural products that are critical in the development of new medicines. Here, we isolated a novel carotenoid from *Arthrobacter* sp. QL17 and characterized its properties. The carotenoid was extracted with methanol, and purified by column chromatography and semi-preparative HPLC. Based on micrOTOF-Q and NMR analyses, the pigment was chemically characterized as 2,2′-((((1E,3E,5E,7E,9E,11E,13E,15E,17E,19E)-3,7,14,18-tetramethylicosa-1,3,5,7,9,11,13,15,17,19-decaene-1,20-diyl)bis(2,2,4-trimethylcyclohex-3-ene-3,1-diyl)) bis(ethan-2-yl-1-ylidene))bi(propane-1,3-diol), and named arthroxanthin. The biological activities of arthroxanthin were evaluated with DPPH, ABTS and MTT assays. Arthroxanthin exhibited excellent radical scavenging properties, as shown for 2, 20-diphenyl-1-picrylhydrazyl (DPPH) and 2,2-n-(3,2-ethyl-benzothiazole-6-sulfonic acid) ammonium salt (ABTS), respectively, with IC50s of 69.8 and 21.5 µg/mL. It also showed moderate anticancer activities against HepG2, Hela, MDAB-231, SW480, and MKN-45 with IC50 values of 107.6, 150.4, 143.4, 195.9, and 145.5 μg/mL, respectively. Therefore, arthroxanthin derived from *Arthrobacter* sp. QL17 may be a potent antioxidant and anticancer agent for food and pharmaceutical use.

## 1. Introduction

Pigments are widely used in food, cloth, painting, cosmetics, pharmaceuticals, and plastics. Due to the synthetic colorants’ hazardous nature, the current exploitations and the prospective of microbial pigments as natural colorants in the food industry are promising. Natural pigments are mainly derived from plants and microorganisms. Plant-derived pigments have long growth cycles which limit their applications at large scales. By comparison, pigments derived from microorganisms have broader application prospects due to the fast growth of microorganisms and low production costs [1]. However, due to the relatively low concentration and the high culture cost of pigments produced by microorganisms, they cannot compete with synthetic pigments economically. Therefore, it is particularly important to obtain strains that can be used in industrial production and can be reduced through strain improvement or the use of low-cost and renewable energy.

The carotenoid group of pigments is widely distributed in plants and microorganisms [2]. The 1204 carotenoids identified from 722 source organisms have been classified as C30, C40, and occasionally C50, depending on the number of carbons in their carotene backbones [3]. Carotenoids exhibit efficiency in treating various diseases, and have antioxidant and anticancer properties [4,5]. A long-term study of antioxidant activity suggested that carotenoids should be considered as a category of dietary antioxidants.

The genus *Arthrobacter*, found in soils, food, water, and plants, produces various carotenoids [6]. Considerable of psychrotrophic and psychrophilic taxa have been isolated from cold environments successfully; among these taxa, Arthrobacter is a comparatively high abundance of the Actinomycetes’ members, with an extensive number of cold-adapted species, such as *A. ruber*, and *A. psychrochitiniphilus*, and *A. glacialis*. Reddy et al. [7] and Gallup et al. [8] isolated two strains of *Arthrobacter* (*A. flavus* and *A. arilaitensis*) that can produce yellow pigments. The absorption spectrum of these yellow pigments had the characteristics of a carotenoid mixture with unknown individual structures. The two pigments (bacterioruberin and decaprenoxanthin) were later identified with ultra-high-performance liquid chromatography-photodiode array detector-mass spectrometry (UPLC-PDA-MS/MS) and reported as C50 carotenoids from *A. agilis* 50cyt and *A. psychrochitiniphilus* 366 [9]. Meanwhile, the rare C50 carotenoids and their glycosylated derivatives are produced by *A. agilis* and *A. bussei* [10,11], whose adaptation to extremely low temperatures may be related to the production of carotenoids.

Mt. Qomolangma (Everest), the highest peak in the world, experiences extremely low temperatures, oligotrophic conditions, and ultraviolet radiation. The annual average temperature is <0 °C. Markus et al. [12] found that carotenoid production increased the resistance to freeze–thaw cycles of heterotrophic bacteria isolated from Antarctic lakes and rivers through repeated freezing, thawing and solar radiation. Thus, a decrease in culture temperature leads to an increase in carotenoid production, which in turn may contribute to membrane stability [13]. Microorganisms living in Mt. Qomolangma may be able to produce novel carotenoids to cope with extreme conditions. Here, we focus on a novel carotenoid obtained from an *A. sp.* QL17 found on Mt. Qomolangma, and on its antioxidant and anticancer activities.

## 2. Materials and Methods

### 2.1. Isolation and Identification of Pigment-Producing Bacteria

Strain QL17 was isolated from soil in the East Rongbuk Glacier on the northern slope of Mount Qomolangma (28.02° N,86.56° E), China, at an elevation of 6350 m. The region is characterized by widespread glaciers and strong surface radiation, with mean annual temperature and mean incoming shortwave radiation ranging from −19 to −3.4 °C and 502 to 635 w/m^2^, respectively. Samples were obtained in May 2019. First, approximately 5 g of soil was dispersed in 25 mL of sterile saline solution and serially diluted with a phosphate-buffered saline (PBS) buffer solution. Then, 100 μL of the suspension was spread on an R2A agar plate and cultivated at 15 °C under aerobic conditions. The isolated strain showing a blue halo around the pink-colored colony on an R2A agar plate was purified and named strain QL17. The pure culture was preserved in glycerol (20%) and stored at −80 °C before use. As described previously [14], isolate strain QL17 was identified by 16S rRNA sequencing using primers F27 and R1492,. The 16S rRNA sequence was compared with the Ezbiocloud database (www.ezbiocloud.net (accessed on 7 July 2020)) to identify the most similar sequences, while phylogenetic trees were constructed using MEGA 7.0 based on the 16S rRNA sequence [15]. The evolutionary distance was calculated by the Maximum Composite Likelihood method. The partial 16S rRNA gene sequence was submitted to the Genbank database and assigned accession number OL471353.

### 2.2. Hydrogen Peroxide (H_2_O_2_) Tolerance Assay

Pure cultures of QL17 were taken out of −80 °C storage and incubated at 15 °C for 7 days. We found in a previous study that strain QL17 had the maximum biomass accumulation at 15 °C. Cells of some well-defined colonies were collected and cultured in 50 mL of R2A liquid medium with shaking (160 rpm) at 15 °C for 3 days. In order to confirm the antioxidant capacity of the strain QL17, cultures in the exponential phase were evaluated for their ability to survive in H_2_O_2_. Cells (OD600 of 1.0) were harvested, washed twice, and resuspended in 0.9% sodium chloride. H_2_O_2_ at different concentrations (50, 100, 150, 200, 250, 300, 400, and 500 mM) was added to diluted samples. After 120 min, the H_2_O_2_ treatment was stopped. Diluted cells were spread on solid R2A plates. All experiments were performed three times. The survival rate is expressed as the percentage of the number of colonies in the treatment group and the number of colonies in the control group (without hydrogen peroxide treatment). Survival rates are expressed as mean values ± standard error of the mean (SEM).

### 2.3. Purification and Structural Identification of the Bioactive Compound

Bacterial colonies were grown in R2A medium in a 1 L conical flask and maintained in a shaking incubator (160 rpm and 15 °C) for 5 days. Bacterial cells were collected following centrifugation at 5000 rpm for 15 min. We used 100% (*v*/*v*) methanol to extract the pigment crude extract in the collected ql17 strain until the strain became colorless. The pigment crude extract was separated and purified on a silica gel column (100–200 mesh, 5 cm × 60 cm, filling height 40 cm) and eluted with gradient mixtures of chloroform–acetone (5:1, 3:1, 1:1, *v*/*v*) and 100% methanol to give four fractions (A–D). We then performed the repeated separation of the fractions (C) with Sephadex LH-20. (CHCl_3_: MeOH, 1:1). C12-1 was purified by semi-preparative high-performance liquid chromatograph (HPLC) with a C18 column (SPQ05-2510WT, High quality & Expert Co., Beijing, China; 10 mm × 250 mm, filling height 250 mm), using ratios of methanol to distilled water of 9:1.

HPLC conditions were as follows. Column type: YMC-Pack ODS-AQ, 250 × 4.6 mm, 5 μm (YMC Co., Tokyo, Japan); flow rate: 0.8 mL/min, mobile phase: methanol and distilled water, 9:1; column temperature: 25 °C; 20 μL of sample was injected per injection and detected by UV-Vis detector at 254 nm.

Chemical characterization of the pure active compound was performed with micrOTOF-Q and NMR analyses. Structural identification of the purified compound was clarified with a DRX-400 spectrometer (Bruker, Rheinstetten, Germany) using spectroscopic techniques for ^1^H,^13^C, HMBC, HSQC, and HHCOSY (400 MHz for ^1^H and ^13^C). The unit of chemical shift is ppm(δ), and residual CHCl3 (δ H 7.26 ppm; δ C 77.0) is used as the internal standard; the coupling constant (J) is in Hz. Moreover, the HMBC and HSQC techniques were also performed to strengthen the results of ^1^H and ^13^C spectroscopic analyses.

### 2.4. Assessment of Radical Scavenging Activity

The DPPH radical scavenging activity assay was modified on the basis of the method previously established by Li et al. [16]. The test method is as follows: mix 100 μL of 0.15 mM DPPH (1,1-diphenyl-2-pyridine hydrazide) solution and 100 μL of the sample solution to be tested thoroughly, and then react in the dark for 30 min and measure the absorbance at 517 nm. The formula for the radical scavenging rate of DPPH is as follows: DPPH radical scavenging activity (%) = [A_1_ − (A_2_ − A_0_)]/A_1_ × 100%. A_1_ is the absorbance of DPPH solution and methanol solution, A_2_ is the absorbance of the DPPH solution and the test sample, and A_0_ is the absorbance of the test sample and methanol without the DPPH solution.

The ABTS radical scavenging activity assay was modified on the basis of the method previously established by Zhu et al. [17]. The test method is as follows: mix 2.45 mmol of potassium persulfate and 7 mmol of ABTS solution. Incubate the mixture in the dark for at least 12 h, then dilute the absorbance of the mixture to 0.7 (at 734 nm) with ethanol before use. (Thermo scientific genesis 30 visible spectrophotometer). Then, 0.5 mL of the test sample was mixed with 4.5 mL of the diluted ABTS solution, and the mixture was incubated for 30 min at room temperature in the dark and we measured the absorbance at 734 nm. The calculation formula of ABTS radical scavenging activity is the same as that of DPPH radical scavenging activity.

The radical scavenging activity of a sample was compared with that of a reference standard β-carotene. Therefore, the antioxidant activity of samples is expressed in terms of equivalence in milligrams of β-carotene. The IC_50_ value was determined as the concentration that caused a 50% reduction in absorbance and was calculated using GraphPad Prism 9.0.

### 2.5. In vitro Cytotoxic Assay

Five human cancer cell lines (HepG-2, SW-1190, MKN-45, Hela, MDAB-231) and one normal human gastric epithelial cell line (GES-1) were tested for antitumor activity, and the cell lines were purchased from the Shanghai Institute of Biochemistry and Cell Biology, Chinese Academy of Sciences. The experimental procedure was as follows: the cells were seeded in RPMI-1640 medium containing 10% (*v*/*v*) fetal bovine serum (FBS), 2 mM glutamine, and 100 units/mL streptomycin–penicillin, and maintained in a humidified atmosphere at 5% CO_2_ and 37 °C.

Cell viability was assessed using the MTT assay [18]. Briefly, cells at a density of 1 × 104 cells/well were seeded in 96-well plates and cultured for 24 h; and then test samples of different concentrations were inoculated into the above cultures for 48 h. Afterwards, 10 μL of MTT (5 mg/mL) solution was added to each well and the incubation was continued for 4 h at 37 °C. Finally, we used a microplate reader to measure the absorbance of the test sample at 490 nm. (Thermo Scientific Multiskan GO, Espoo, Finland).

### 2.6. Data Processing and Statistical Analysis

Differences between the treatments were determined based on analysis of a Student’s *t*-test (nonparametric tests-unpaired) using GraphPad Prism version 9.0, and differences were considered statistically significant at *p* < 0.05. Chemical structures were drawn and exact mass values were calculated using ChemDraw Ultra version 12.0.2. All graphs were produced using GraphPad Prism version 9.0 and origin 2019.

## 3. Results and Discussion

### 3.1. Growth and Characterization of Arthrobacter sp. QL17

**Figure 1:**
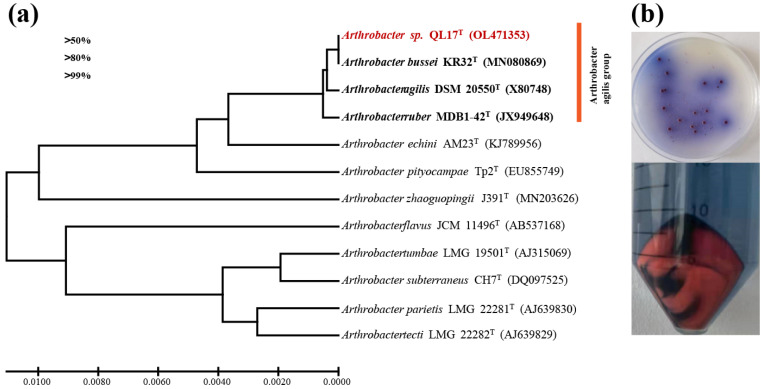
(**a**) A phylogenetic tree based on 16S rRNA gene sequence, and (**b**) colonies on R2A medium and liquid culture of *Arthrobacter* sp. QL17.

A red bacterium designated as strain QL17 was selected from 93 bacterial strains isolated from Mount Qomolangma. The almost-complete 16S rRNA gene sequence (1532 bp) of QL17 was determined and revealed that the strain QL17 represented a member of the ‘*A. agilis* group’ (Figure 1a). Indeed, the 16S rRNA gene sequence of the strain QL17 showed a 99.93, 99.79, and 99.71% similarity with *A. bussei* KR32, *A. agilis* DSM 20550, and *A. ruber* MDB1-42, respectively. The results showed that strain QL17 was closely related to *A. bussei* (identity of 16S rRNA gene, 99.93%). The best growth and pink coloration of strain QL17 were observed in R2A medium at 15 °C for subsequent experiments (Figure 1b).

### 3.2. Hydrogen Peroxide (H_2_O_2_) Stress Tolerance of A. sp. QL17

**Figure 2:**
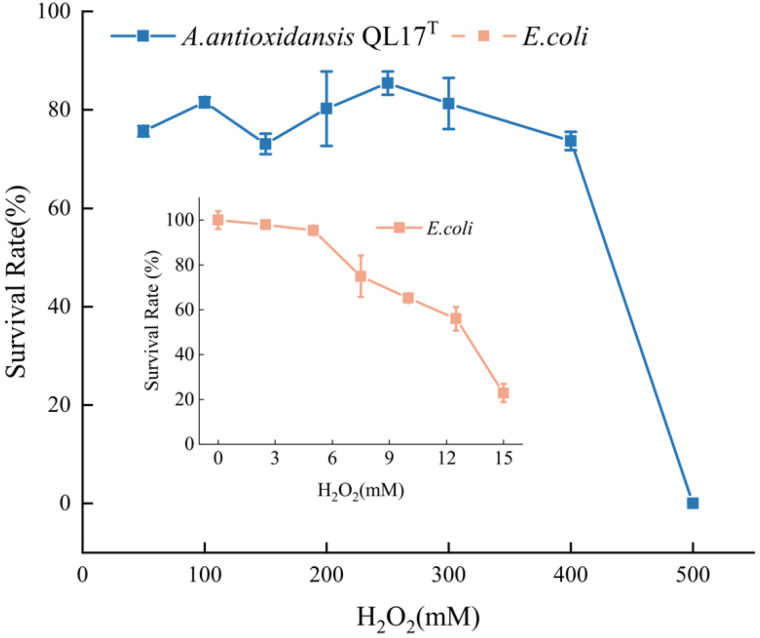
Survival rate of QL17 and *E. coli* stressed by the H_2_O_2_. Error bars represent standard deviation of the mean (*n* = 3); *p* < 0.05.

Bacteria can produce reactive oxygen species under hydrogen peroxide stress, which in turn damage bacterial cell membranes, proteins and DNA. To avoid this, microbial pigments delay or inhibit cellular damage by donating electrons to a free radicals and neutralizing them via their free radical scavenging properties [1]. Therefore, most microorganisms that can produce pigments have robust antioxidant activity. Our results showed that QL17 survival in 400 mM H_2_O_2_ ranged from 73.1 to 85.4% of colonies. The strain was inhibited at 500 mM H_2_O_2_. In another study, the survival rate of *Escherichia coli* was only 22.9% in a 15 mM hydrogen peroxide treatment (Figure 2). Strain QL17 appears to have a more powerful antioxidant activity when compared to *E.coli*, indicating that the pigment production of bacteria provides some protection for cells undergoing oxidative stress. This particular property is primarily responsible for the coloration and the ability of many of these compounds to interact with free radicals and singlet oxygens, and act as effective antioxidants [19].

### 3.3. Purification and Structural Identification of Antioxidants Produced by Strain QL17

**Figure 3:**
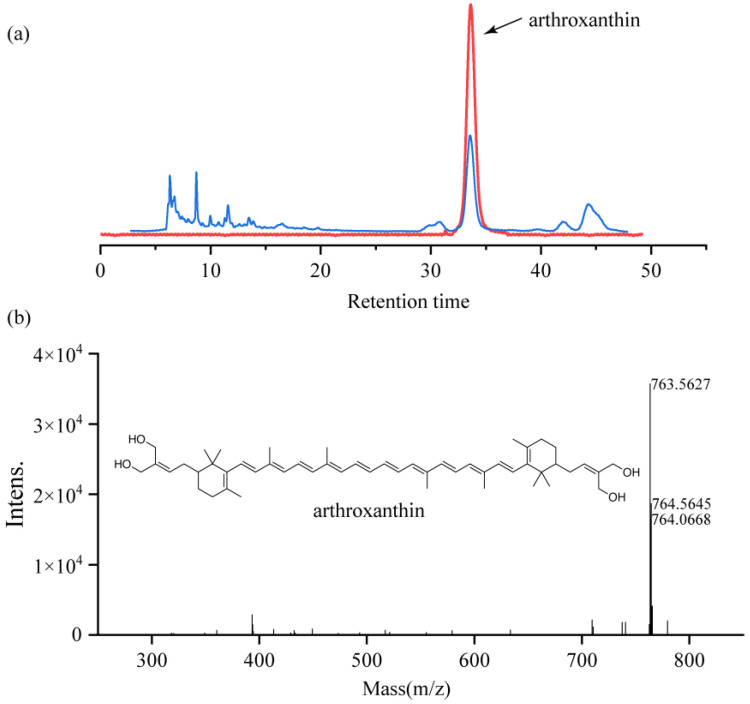
Purification and structural identification of arthroxanthin produced by QL17. (**a**) HPLC analysis of arthroxanthin. (**b**) MicrOTOF-Q analysis of arthroxanthin.

The purified fractions (C) exhibited a sharp single peak at a retention time of 34 min (Figure 3a). The final yield of the highly purified pigment was 8 mg from a 314 mg solvent-extracted sample.

Compound C12-1 was isolated as a red amorphous powder and its molecular formula was established as C52H74O4 by HR-ESI-MS at m/z 763.5627 [M + H]+ (Figure 3b), indicating 16° of unsaturation. The 13C NMR spectrum exhibited 52 carbon resonances, containing ten methyl groups, ten sp3 methylenes, two sp3 methines, twenty-eight olefinic carbons, and two quaternary sp3 carbons. The 1H NMR spectroscopic data (Table 1) indicated olefinic protons at δH 6.68, 6.41, 6.29, 5.48, methyls at δH 1.98, 1.93, and 1.21, and three signal characteristic of a methylene group at δH 3.62, 1.99, and 1.55. The above information, along with the extensive analysis of ^1^H-^1^H COSY, HSQC, and HMBC spectroscopic data led to the establishment of the planar structure of compound C12-1(Supplementary Figures S1–S5). Therefore, the novel pure, red amorphous powder was identified as a carotenoid, and subsequently named arthroxanthin. The chemical structure of arthroxanthin belongs to the ‘C50 carotenoid family’ (Figure 3b). Based on carotenoid database statistics (http://carotenoiddb.jp (accessed on 5 August 2021)), bacteria can synthesize nearly all C45, some of C30, and C50 carotenoids. Currently, more than 95% of the natural carotenoids that have been discovered are based on the symmetrical C40 phytoene backbone, with only small amounts of C30 or even less C50 carotenoids found [20].

Carotenoids act as non-enzymatic antioxidants and may be involved in bacterial resistance to environmental stress. Such as a colorless mutant isolated from the wild-type *Deinococcus radiodurans* was 100 times more sensitive to H_2_O_2_ treatment at a concentration of 50 mM than the wild-type strain [21]. Therefore, the synthesis of carotenoids in strain QL17 may be a partial adaptive feature that safeguards the survival of this bacterium in the extreme environment of Mt. Qomolangma.

### 3.4. The Radical-Scavenging Activity of Arthroxanthin

**Figure 4:**
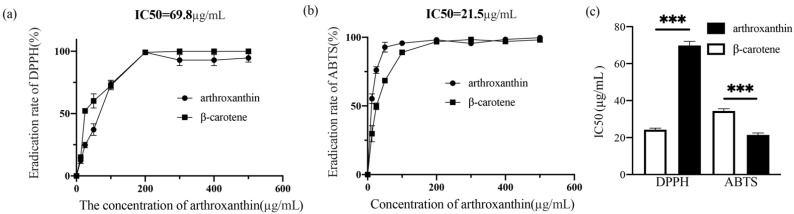
(**a**) DPPH radical scavenging activity of arthroxanthin. (**b**) ABTS radical scavenging activity of arthroxanthin. (**c**) IC_50_ values of β-carotene and arthroxanthin in different radical scavenging activity assays. *** *p* < 0.001 compared with β-carotene.

DPPH and ABTS are substrates for measuring the radical scavenging activity of compounds in vitro, which can characterize the free radical scavenging activity and antioxidant potential of compounds [22]. Most carotenoids have long-chain conjugated polyenes, giving them a unique yellow to red coloration and antioxidant activities, such as radical scavenging and singlet oxygen quenching [23]. Sahli et al. [24] found that the ability of C50 carotenoids produced by halophilic Archaea to scavenge DPPH free radicals was significantly higher than that of β- Carotene. Concentration-dependent assays were carried out with arthroxanthin and β-carotene. Arthroxanthin possessed significant scavenging activity against DPPH and ABTS radicals, indicating that it has an antioxidant effect; the scavenging effect increased with increasing concentration (Figure 4a,b). Arthroxanthin exhibited 73.4% DPPH scavenging activity at 100 μg/mL and 76.1% ABTS scavenging activity at 25 μg/mL. The DPPH and ABTS scavenging activity were characterized by the IC50 value. The IC50 value of DPPH and ABTS scavenging activity was 69.8 and 21.5 μg/mL, respectively. The scavenging activity of β-carotene and arthroxanthin differed significantly (*p* < 0.001, Student’s *t*-test; Figure 4c). The ability of 1 μg of arthroxanthin to scavenge DPPH and ABTS radical was equal to 2.9 and 0.6 μg of β-carotene, respectively. The antioxidant capacity of this novel pigment (arthroxanthin) is comparable to well-known antioxidants such as β-carotene. These data suggest that *Arthrobacter* sp. QL17 is a likely source of bacteria-based antioxidant production.

### 3.5. Cytotoxic Activities of Arthroxanthin

**Figure 5:**
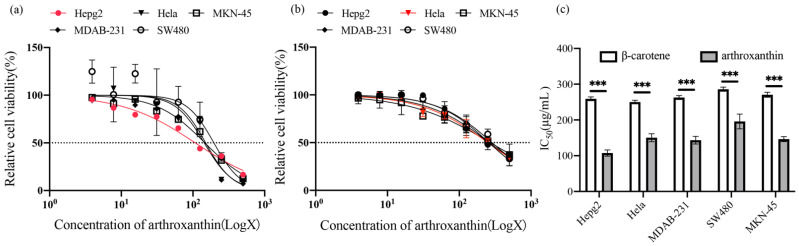
Effect of arthroxanthin on the cell viability of various cancer types. (**a**) Cancerous cells were incubated with different concentrations of arthroxanthin (0–500 µg/mL) for 48 h. Then, cell viability (%) was assessed by an MTT assay. (**b**) Cancerous cells were incubated with different concentrations of β-carotene (0–500 µg/mL) for 48 h. (**c**) IC_50_ values of β-carotene and arthroxanthin against cancerous cells at 48 h. *** *p* < 0.001 compared with β-carotene.

Recently, there has been an increasing interest in studying the effects of natural products on human health, especially those compounds that have been shown to be effective against cancer [25,26]. For instance, lycopene inhibits the development of squamous cell carcinomas of the stomach [27] and induces apoptosis in smoke-induced lung cancer [28]. Some carotenoids also have the potential to prevent cancer; as among them, fucoxanthin in brown algae and neoxanthin in spinach can fight prostate cancer cells [29]. Faraone et al. [30] found that astaxanthin can play an anticancer role in melanoma, gastric carcinoma, or colorectal cancer, and enhance the effectiveness of traditional chemotherapy drugs. Carotenoids inhibit cancer cell proliferation mainly by inducing apoptosis, increasing gap-junction communication, and arresting the cell cycle [31]. Because of the remarkable antioxidant activity described above, the red pigment (arthroxanthin) of QL17 was examined against five representative human malignant tumors and the normal GES-1 cells in vitro (Figure 5).

In this study, a dose effect was the antiproliferative activity of arthroxanthin against different cancer cell lines increased with arthroxanthin concentration. Arthroxanthin exhibited cytotoxic activity against five cancer cell lines with IC50 values <200 μg/mL (Figure 5). Specifically, arthroxanthin was most potent toward Hepg2, followed by MDAB-231, MKN-45, Hela, and SW480 with IC50 values of 107.6, 150.4, 143.4, 195.9, and 145.5 μg/mL, respectively, after 48 h of treatment (Figure 5a). This indicates a moderate antitumor effect on the five representative human malignant tumors. Furthermore, arthroxanthin displayed greater cytotoxicity towards Hepg2, MDAB-231, MKN-45, Hela, and SW480 in vitro than β-carotene with IC50 values > 200 μg/mL (Figure 5b). Statistical analysis revealed a significant difference in anticancer activity between β-carotene and arthroxanthin (*p* < 0.001, Student’s *t*-test; Figure 5c) against different cancer cell lines. In conclusion, arthroxanthin potently inhibited various cancer cell types, with a preference for Hepg2. Previous studies have shown that carotenoids, such as neoxanthin, provide adequate protection against stress-mediated cytotoxicity by blocking apoptosis and activating the intracellular antioxidant defense system [32].

## 4. Conclusions

In this study, a red pigment produced by a bacterial strain *Arthrobacter* sp. QL17 isolated from Mt. Qomolangma was identified for the first time as a carotenoid arthroxanthin. The H_2_O_2_ tolerance assay revealed a presence in strain QL17 of powerful antioxidant activity. Arthroxanthin was extracted with methanol, and purified with column chromatography and semi-preparative HPLC. The compound was determined to be a carotenoid (arthroxanthin) using micrOTOF-Q and NMR and its pigment was chemically characterized. In addition, arthroxanthin derived from strain QL17 exhibited radical scavenging (e.g., DPPH and ABTS) and potentially anticancer activity which may be a promising source of a natural pigment for use in the food industry and a bioactive compound for use in the pharmaceutical industry.

However, since biological activity determination was completed in vitro in this study, a higher non-physiological concentration was used, and the compound’s in vivo effect is uncertain. In addition, the diversity of the biological activities of arthroxanthin and its biosynthetic mechanism in Arthrobacter sp. QL17 needs further study.

## Figures and Tables

**Table 1:** NMR spectroscopic data for the arthroxanthin.

Position	*δ*_H_	*δ*_C_	Position	*δ*_H_	*δ*_C_
1,1′	3.62 *	71.2,72.9	14,14′	6.68	131.6
2,2′		138.9	15,15′	6.68	129.6
3,3′	5.48	124.6	16,16′		138.3
4,4′	1.92	27.2	17,17′	6.41	125.3
5,5′	1.77	42.7	18,18′	6.68	133.1
6,6′	1.55	29.7	19,19′	6.68	130.5
7,7′	1.99	29.9	20,20′		138.2
8,8′		134.7	21,21′	6.29	125.3
9,9′		133.3	22,22′	6.29	131.6
10,10′		29.4	23,23′	6.29	129.6
11,11′	1.21	24.5	24,24′	1.98	13.1
12,12′	1.21	24.5	25,25′	1.98	13.0
13,13′	1.93	13.3			

The assignments were based on ^1^H-^1^H COSY, HSQC, and HMBC experiments. * this signal peak is determined by the HSQC spectrum.

## Data Availability

Data is contained within the article.

## Supplementary Materials

The following are available online at https://www.mdpi.com/article/10.3390/antiox11081493/s1, Figures S1–S5: The ^13^C NMR, ^1^H NMR, ^1^H-^1^HCOSY, HMBC, and HSQC spectrum of compound arthroxanthin.
