# Supplementary material for: Purification, Identification, and Properties of a Novel Carotenoid Produced by Arthrobacter sp. QL17 Isolated from Mount Qomolangma
Source: Antioxidants (Basel). 2022 Jul 29;11(8):1493. doi: 10.3390/antiox11081493 (PMC9404904; doi:10.3390/antiox11081493)
Supplement: Supplementary file 1 [file antioxidants-11-01493-s001.zip › antioxidants-1827126-supplementary.pdf]

Purification and characterization of a new carotenoid produced by *Arthrobacter antioxidansis* QL17 isolated in Mount Qomolangma.

Xue Yu[1,2,4], Kan Jiang[5], Wei Zhang[1,2]*, Shuqing Dong[6], Yujie Wu[2,3,4], Gaosen Zhang[2,3], Shiyu Wu[2], Tuo Chen[2,3], & Guangxiu Liu[1,2*]

Corresponding author: Wei Zhang & Guangxiu Liu*

E-mail address: ziaoshen@163.com, Guangxiu Liu, liugx@lzb.ac.cn;

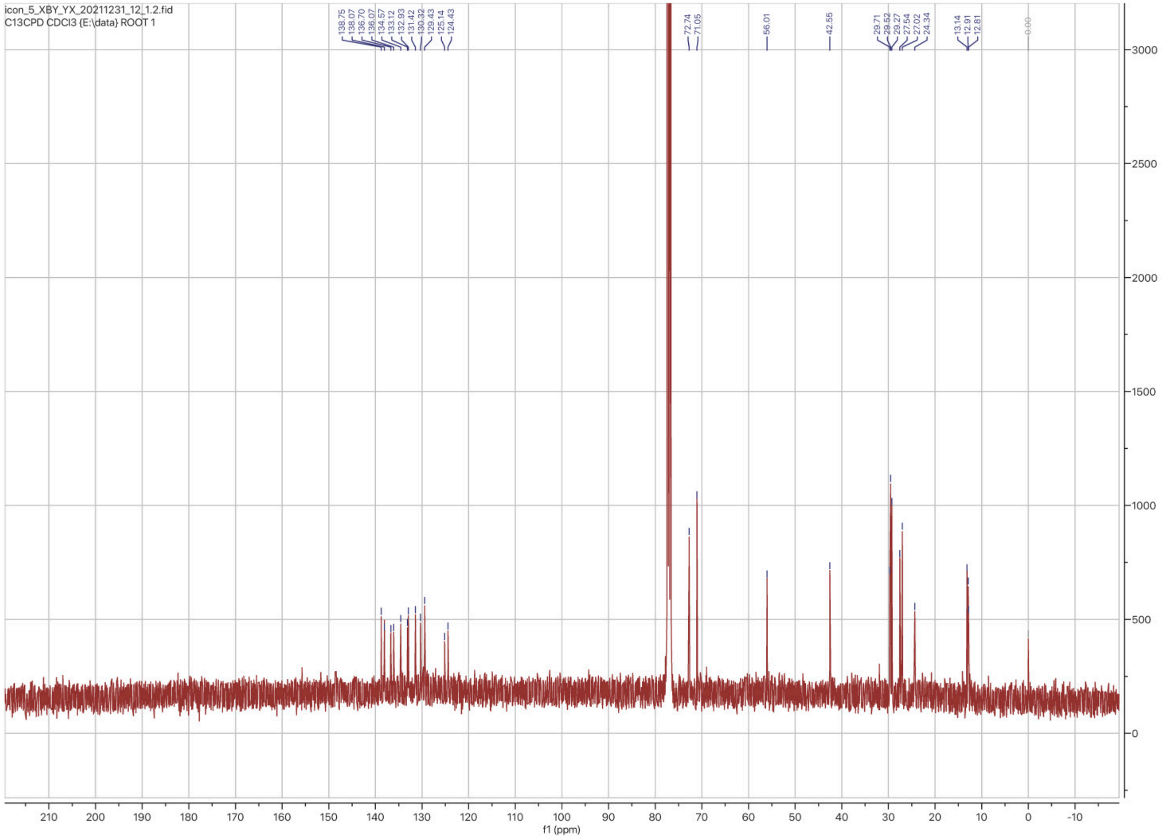

Figure S1. ^{13}C NMR spectrum of arthroxanthin in $CDCl_3$ (100 MHz).

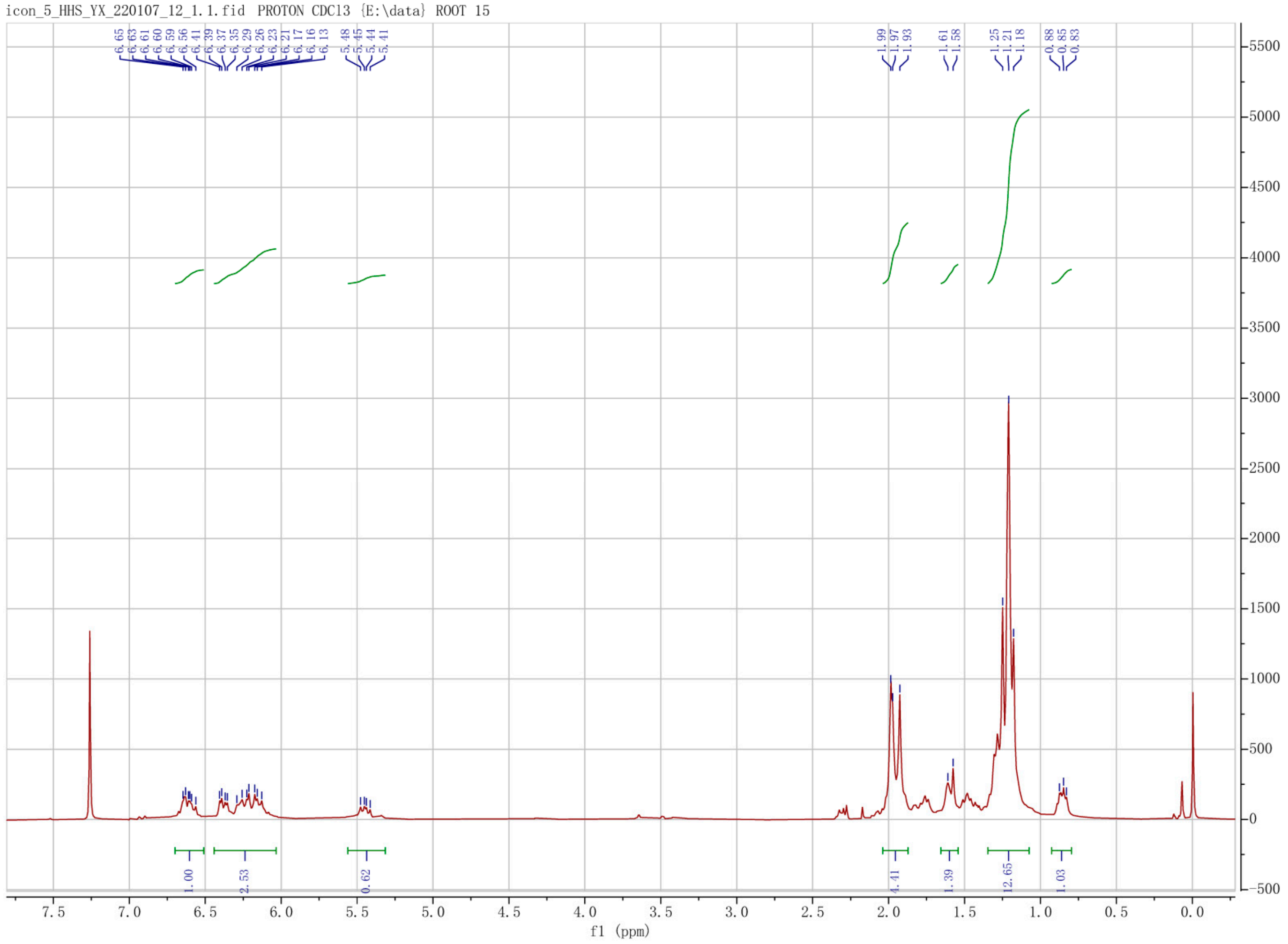

Figure S2. ^{1}H NMR spectrum of arthroxanthin in $CDCl_3$ (400 MHz).

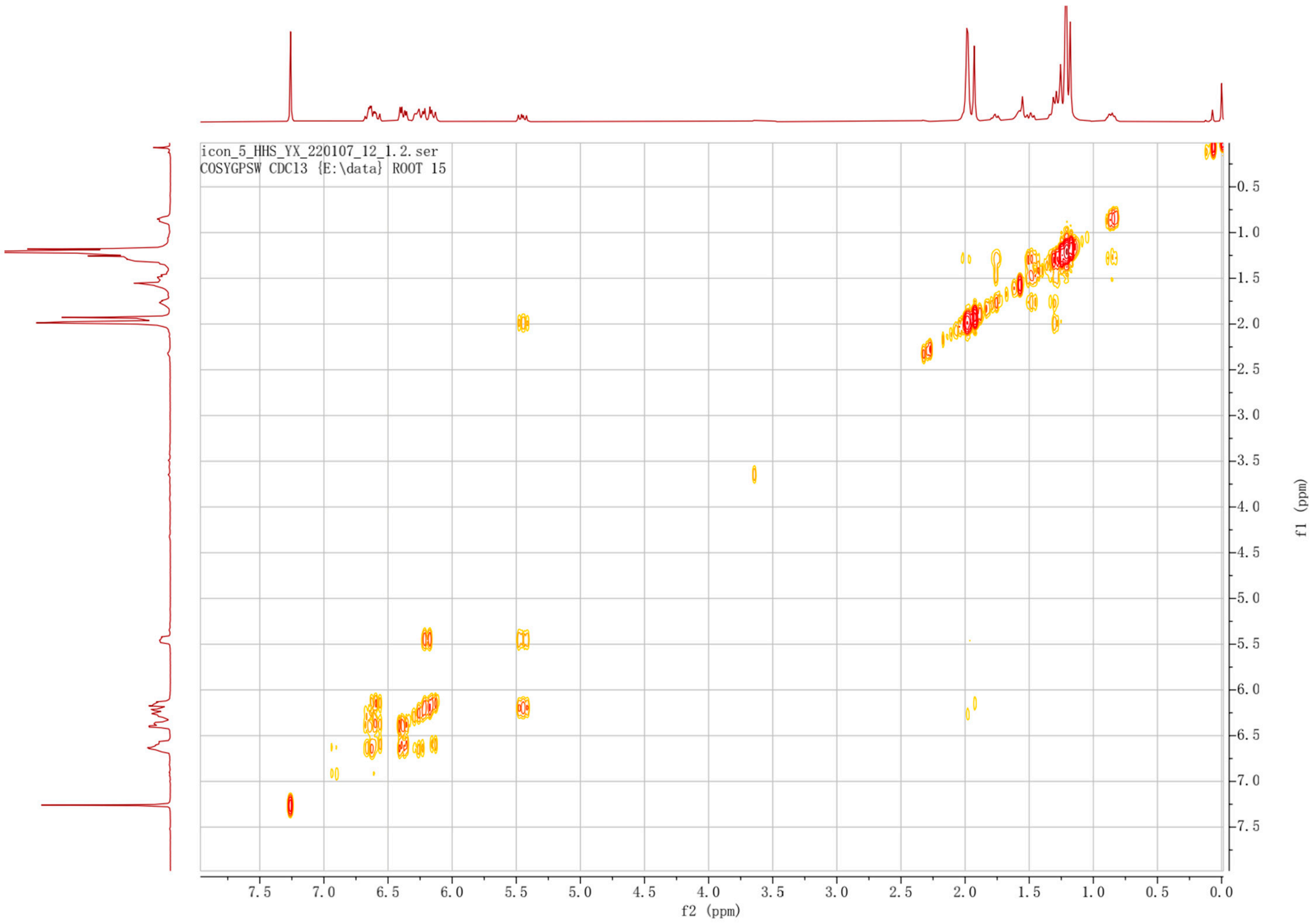

Figure S3. ^{1}H-^{1}HCOSY spectrum of arthroxanthin in $CDCl_3$

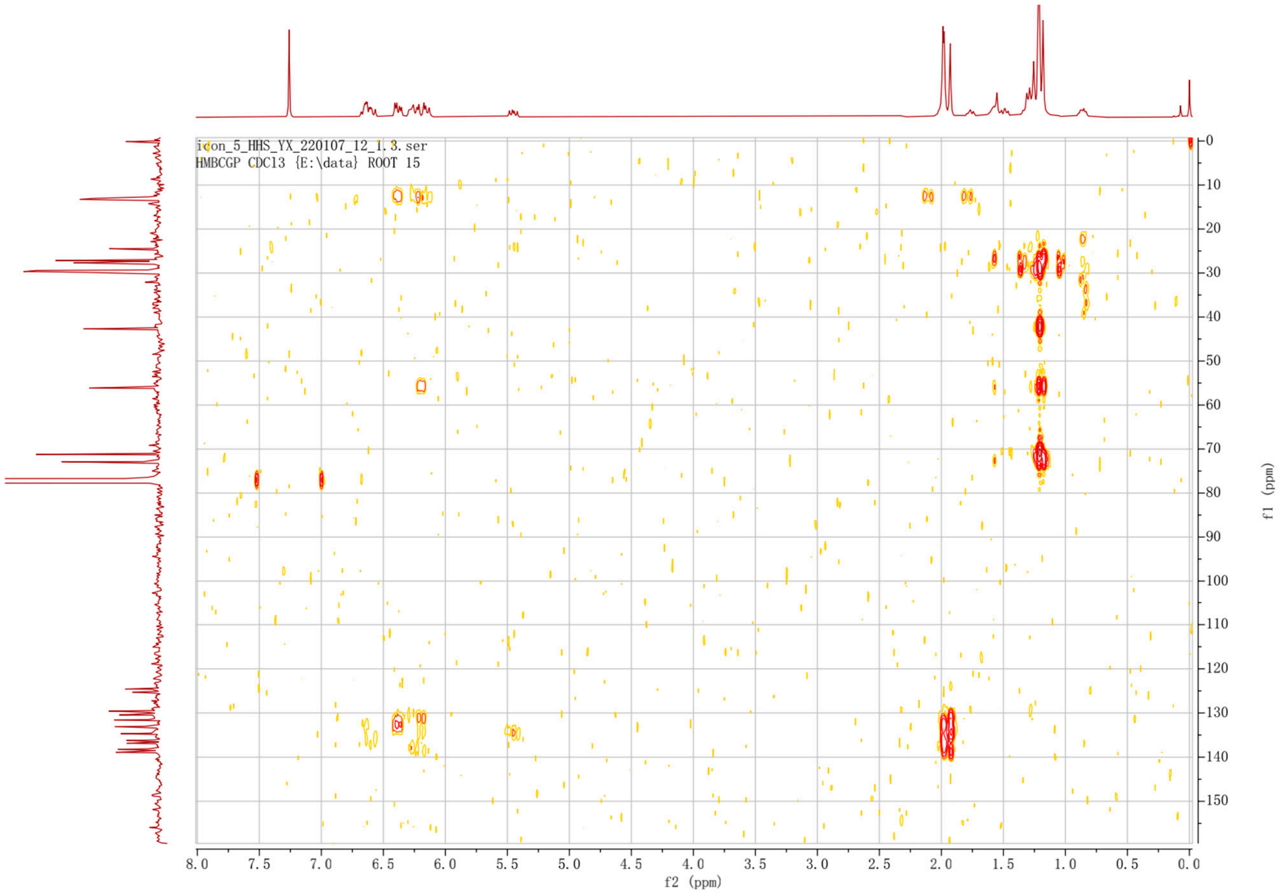

Figure S4. HMBC spectrum of arthroxanthin in $CDCl_3$.

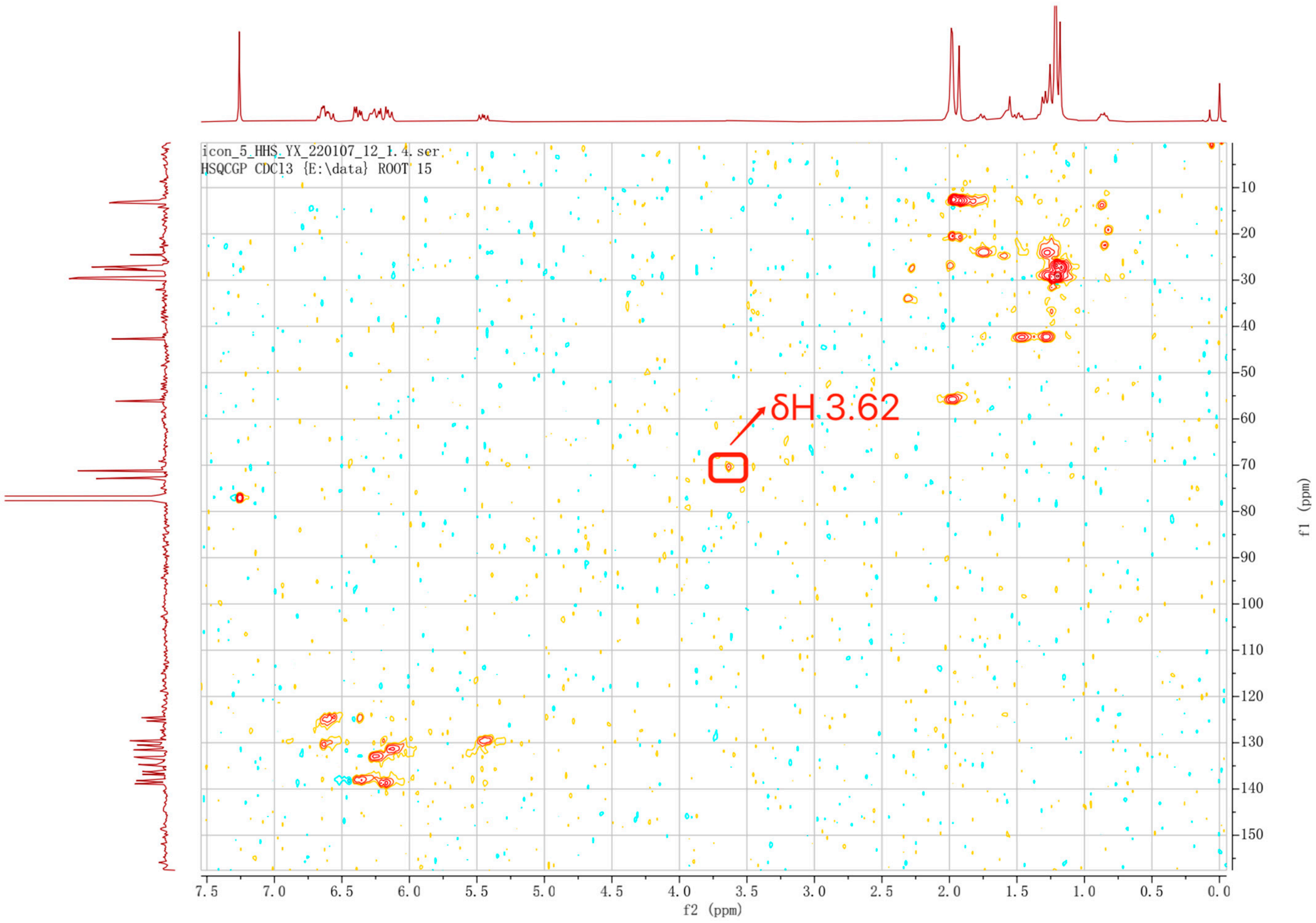

Figure S5. HSQC spectrum of arthroxanthin in $CDCl_3$.
